# Supplementary material for: Lead in traditional eyeliners: An investigation into use and sources of exposure in King County, Washington
Source: PLOS Glob Public Health. 2025 Jun 25;5(6):e0004643. doi: 10.1371/journal.pgph.0004643 (PMC12193921; doi:10.1371/journal.pgph.0004643)
Supplement: S1 Appendix — (DOCX) [file pgph.0004643.s001.docx]

**Supplementary Material**

**Appendix A: Sample acquisition**

*A.1 Eyeliners provided by the community*

From 2022 to 2023, King County community members brought personal items to events to be analyzed by Public Health – Seattle & King County (PHSKC) staff for lead content either on-site by X-ray fluorescence analysis or by a commercial laboratory. Staff collected information on the product from participants and any labels present on the item. Information collected included product description, brand name, manufacturer, country of manufacture, purchase location, and batch number (if applicable). Eye cosmetics included eyeliners, eyeshadows, and mascaras, as well as traditional eyeliners such as kajal, kohl, and surma.

Samples were also collected via collaboration with the Afghan Health Initiative (AHI), a grass-roots non-profit organization in Washington state (WA) founded by current and former immigrants and refugees from Afghanistan. Due to the high prevalence of Afghan children with blood lead levels (BLLs) above the Centers for Disease Control and Prevention’s (CDC) recommended blood lead reference value (BLRV), the Hazardous Waste Management Program (Haz Waste Program) collaborated with AHI to research potential lead exposures and effective interventions. AHI visited the homes of 32 families between June 1 and September 30, 2023, to administer questionnaires and collect traditional eyeliners for testing. AHI staff also collected eyeliners from community members during a community forum on lead in traditional eyeliners on November 18, 2023. Consequently, 21 products were provided to the Haz Waste Program for testing. AHI staff collected information from participants on the name of the eyeliner, where it was purchased, and whether it was homemade.

In 2022, PHSKC investigators found four traditional eyeliners that contained hazardous lead concentrations during in-home visits in King County, WA. These samples were then transferred to the Haz Waste Program researchers for further analysis.

Two additional products were recommended for testing by other community members. One was recommended by the Multnomah County Health Department in Portland, Oregon, and another was recommended by a PHSKC employee. Additionally, a PHSKC employee provided kohl samples purchased in Saudi Arabia.

*A.2 Eyeliners purchased by researchers*

Some Afghans were aware of the dangers of lead in traditional eyeliners and were using cosmetics they deemed to be “safer alternatives.” They often purchased these products from local King County grocery stores or via online retailers such as Amazon. AHI provided the Haz Waste Program with a list of these products, which they subsequently purchased from Amazon for testing (when available). Products included eyeshadow pallets, eyeliners, and mascaras. To assess the availability of hazardous products from online retailers other than Amazon, products were purchased on eBay and Etsy. Search terms included “kajal”, “kohl,” and “surma“ because they were suspected to contain lead based on prior data gathered during in-home investigations and community product lead testing events which consistently identified traditional eyeliners with high lead concentrations labeled as “kajal”, “kohl”, and “surma.” The products identified in the top search results were purchased as these were the most popular items on the site.

Traditional eyeliners purchased online often used multiple eyeliner terms in their advertising; however, when categorizing traditional eyeliners, we only used the term displayed on the physical packaging.

**Appendix B: Laboratory analysis methods**

We endeavored to collect at least one gram (1 g) of sample to prevent matrix interference during laboratory analysis. Before sending products for chemical analysis, a 210-millimeter (mm) spatula was used to transfer eyeliner product from its original container into a plastic sample bag.

*B.1 Analysis Method using Inductively Coupled Plasma-Mass Spectrometry (ICP-MS)*

Samples sent to the University of Washington’s Environmental Health Laboratory and Trace Organics Analysis Center (Seattle, Washington) (UW EHL TOAC) were analyzed using the EHLSOP-07 method, which is based on the United States (US) Environmental Protection Agency’s (EPA) 6020a Rev.1 2007 method [1], via an Agilent 7900 SPS4 autosampler interfaced with an Agilent 7900 CE ICP-MS system. Nitric acid (HNO_3_), hydrochloric acid (HCL), hydrofluoric acid (HF), and tetrafluoroboric acid (HBF_4_), were added to samples, and samples were placed in a microwave to digest (Personal email communication between Aesha Mokashi (Haz Waste Program) and Shar Samy (UW EHL TOAC), October 2, 2024). Samples were then placed in the ICP-MS systems for analysis. Reagents and method blanks were run with each batch, and lead concentrations in samples were blank corrected. Two sub-aliquots per sample were analyzed to demonstrate homogeneity/heterogeneity. Matrix spike recovery was performed on one sample. The relevant detection limit was reported as the reporting limit (RL). The lowest detectable calibrant used to determine the instrument’s detection limit was 0.001 parts per million (ppm) or 1 part per billion (ppb), and the sample extract volume for our batch of samples was 50 milliliters (mL). For a 1 g sample, the typical RL was 0.05 ppm. Eleven products were analyzed using this method, none of which were <RL (Table B.1).

*B.2 Analysis Method using Inductively Coupled Plasma Optical Emission Spectroscopy (ICP-OES)*

Samples sent to NVL Laboratories, Inc. (Seattle, Washington) were analyzed using the Consumer Product Safety Commission Directorate (CPSC)-CH-E1002-08 (Children’s non-metal products) method [2] via an ICP-OES system. Samples were prepared with deionized water and digested using high-purity concentrated HNO_3_ and microwave. They were then diluted and placed in the ICP-OES system for analysis. Each sample set was analyzed with a blank to check for background levels. Blank correction was not necessary because none of the blanks contained lead levels above the RL. The lowest detectable calibrant used to determine the instrument’s detection limit was 0.2 ppm, and the typical sample extract volume for this method was 20 mL. For a 1 g sample, the typical RL was 4 ppm (Personal email communication between Aesha Mokashi (Haz Waste Program) and Shalini Patel (NVL Laboratories), September 3, 2024). NVL Laboratories reported that RL was based on the lowest concentration analyzed daily which can be quantified with a certain degree of confidence and can be reproducible within an established acceptable range (Personal email communication between Aesha Mokashi (Haz Waste Program) and Nick Ly (NVL Laboratories), March 14, 2024). For samples with lead concentrations <RL, laboratory-reported lead concentrations were included in the analysis when available. However, samples with lead concentrations reported as 0 ppm were included using imputations. Sixty-eight products were analyzed using this method, and 53 samples were <RL (Table B.1).

*B.3 Analysis Method using Graphite Furnace Atomic Absorption Spectrometry (GFAA)*

Samples sent to NVL Laboratories were analyzed using the EPA 7010 method [3] via a GFAA system. Samples were prepared with deionized water and digested using high-purity concentrated HNO_3_ and microwave. They were then diluted and placed in the GFAA system for analysis. Each sample set was analyzed with a blank to check for background levels. Blank correction was not necessary because none of the blanks contained lead levels above the RL. The lowest detectable calibrant used to determine the instrument’s detection limit was 10 ppb (0.01 ppm), and the typical sample extract volume was 20 mL (Personal email communication between Aesha Mokashi (Haz Waste Program) and Shalini Patel (NVL Laboratories), September 3, 2024). For a 1 g sample, the typical RL was 0.2 ppm. RL at NVL Laboratories is based on the lowest concentration analyzed daily which can be quantified with a certain degree of confidence and can be reproducible within an established acceptable range (Personal email communication between Aesha Mokashi (Haz Waste Program) and Nick Ly (NVL Laboratories), March 14, 2024). For samples with lead concentrations <RL, laboratory-reported lead concentrations were included in the analysis when available. However, samples with lead concentrations reported as 0 ppm were included using imputations. Sixty-six products were analyzed using this method, and eight samples were <RL (Table B.1).

**Table B.1**

Lead concentration in eyeliners descriptive statistics by different lab techniques. *

| Analysis method | Instrument | n | RL (ppm) | % samples <RL | % samples 0 ppm | 1st quartile (ppm) | Median [range] (ppm) | 3rd quartile (ppm) | Mean +/- SD (ppm) |
| --- | --- | --- | --- | --- | --- | --- | --- | --- | --- |
| All methods | -- | 145 | -- | 42% | 22% | 2.8 | 3.2 [<0.2 - 840,000] | 113.1 | 102,370 +/- 225,007 |
| EPA 6020a Rev.1 2007 | ICP-MS^a^ | 11 | 0.05 | 0% | 0% | 241 | 391,500 [10 - 785,500] | 608,000 | 17,658 +/- 118 |
| CPSC-CH-E1002-08 (Children’s non-metal products) | ICP-OES^b^ | 68 | 4 | 78% | 47% | 0.47 | 0.04 [<4- 840,000] | 12.2 | 69,891 +/- 203,304 |
| EPA 7010 | GFAA^c^ | 66 | 0.2 | 12% | 3% | 0.80 | 5.0 [<0.2 - 720,000] | 170 | 95,188 +/- 208,289 |
| RL, analytical method reporting limit; ppm, parts per million; SD, standard deviation; ICP-MS, Inductively Coupled Plasma-Mass Spectrometry; ICP-OES, Inductively Coupled Optical Emission Spectroscopy; GFAA, Graphite Furnace Atomic Absorption Spectrometry.  ^a^ICP-MS measurements were conducted by the University of Washington’s Environmental Health Laboratory and Trace Organics Analysis Center, Seattle, WA.  ^b^ICP-OES measurements were conducted by NVL Laboratories, Seattle, WA. Each sample had a different RL due to differences in weight and matrix. The range for samples <RL was <4 to <160.  ^c^GFAA measurements were conducted by NVL Laboratories. Each sample had a different RL due to differences in weight and matrix. The range for samples <RL was <0.2 to <1.1.  *Note: For left-censored data, imputations were used to calculate descriptive statistics. | | | | | | | | | |

**References**

1. United States Environmental Protection Agency. Method 6020A Inductively Coupled Plasma-Mass Spectrometry. 2014 July.

2. United States Consumer Product Safety Commission Directorate for Laboratory Sciences Division of Chemistry. United States Consumer Product Safety Commission Test Method: CPSC-CH-E1002-08.3 Standard Operating Procedure for Determining Total Lead (Pb) in Nonmetal Children's Products, Revision. 2012 Nov 15.

3. United States Environmental Protection Agency. Method 7010 Graphite Furnace Atomic Absorption Spectrophotometry. 1998 January.
