# Supplementary material for: Lead in traditional eyeliners: An investigation into use and sources of exposure in King County, Washington
Source: PLOS Glob Public Health. 2025 Jun 25;5(6):e0004643. doi: 10.1371/journal.pgph.0004643 (PMC12193921; doi:10.1371/journal.pgph.0004643)
Supplement: S2 Appendix — (DOCX) [file pgph.0004643.s002.docx]

**Regression analyses & reporting parameters**

**S2.1**

Reporting parameters for all Kruskal Wallis tests.

| Variable | Chi-squared | df | p-vlaue |
| --- | --- | --- | --- |
| Kajal v Kohl v Surma | 12.33 | 2 | 0.002 |
| Production country  (All eyeliners) | 17.98 | 2 | <0.001 |
| Production country  (Traditional eyeliners) | 2.99 | 2 | 0.22 |

**S2. 2**

Reporting parameters for all Wilcoxon Rank Sum tests

| Variable | W | p-value |
| --- | --- | --- |
| Kohl v Kajal | 472 | 0.01506 |
| Surma v Kajal | 472 | <0.001 |
| US/EU v Other  (Traditional eyeliners) | 233 | 0.17 |
| US/EU v Afghan  (Traditional eyeliners) | 71 | 0.019 |
| Afghan v Other  (Traditional eyeliners) | 212 | 0.17 |
| US/EU v Other  (All eyeliners) | 1425 | 0.0011 |
| Afghan v U/EU  (All eyeliners) | 285 | <0.001 |
| Afghan v Other  (All eyeliners) | 388.5 | 0.016 |
| Cream v Powder  (Traditional eyeliners) | 1241.5 | <0.001 |
| Cream v Powder  (All eyeliners) | 3056 | <0.001 |
| Homemade v Manufactured  (Traditional eyeliners) | 266 | 0.22 |
| Homemade v Manufactured  (All eyeliners) | 265.5 | 0.99 |
| YesLabel v NoLabel  (Traditional eyeliners) | 194 | 0.26 |
