# Supplementary material for: Lead in traditional eyeliners: An investigation into use and sources of exposure in King County, Washington
Source: PLOS Glob Public Health. 2025 Jun 25;5(6):e0004643. doi: 10.1371/journal.pgph.0004643 (PMC12193921; doi:10.1371/journal.pgph.0004643)
Supplement: Attachment — Submitted filename: Mokashi_Response letter_3-24-25.docx [file pgph.0004643.s005.docx]

Editor:

Journal Requirements:
1. Please provide separate figure files in .tif or .eps format.
For more information about figure files please see our guidelines:
[https://journals.plos.org/globalpublichealth/s/figures](https://urldefense.com/v3/__https:/journals.plos.org/globalpublichealth/s/figures__;!!K-Hz7m0Vt54!icTSzNb0o9aYhAkglhEwP-7v-A1z6TuAn1UAPHRWL0lFoc9ffqPLwCAXMp3khupIg7bJT4z-wAZ0eEof$)
[https://journals.plos.org/globalpublichealth/s/figures#loc-file-requirements](https://urldefense.com/v3/__https:/journals.plos.org/globalpublichealth/s/figures*loc-file-requirements__;Iw!!K-Hz7m0Vt54!icTSzNb0o9aYhAkglhEwP-7v-A1z6TuAn1UAPHRWL0lFoc9ffqPLwCAXMp3khupIg7bJT4z-wCR8u_Hy$)

- **Author Response**:
- Thank you for the helpful links. We have edited the photo so that it is 300 dpi, is 2250 pixels x 1590 pixels, and is in .tif format.

2. We do not publish any copyright or trademark symbols that usually accompany proprietary names, eg (R), (C), or TM (e.g. next to drug or reagent names). Please remove all instances of trademark/copyright symbols throughout the text, including ™ on page 11.

- **Author Response**:
- We have removed the trademark symbols on page 11. Thank you.

3. Figure 1: Please confirm (a) that you are the photographer; or (b) provide written permission from the photographer to publish the photo(s) under our CC-BY 4.0 license.

- **Author Response**:
- Aesha Mokashi and Katie Fellows are the photographers. We have credited them in the Fig 1 legend.

4. Figure 1 contains branding/a logo. We are not permitted to publish this under our CC-BY 4.0 license, even with permission. We ask that you please remove or replace it.

- **Author Response**:
- Thank you for your comment. We have removed the images in Figure 1 with branding/logo.

5. Please review your reference list to ensure that it is complete and correct. If you have cited papers that have been retracted, please include the rationale for doing so in the manuscript text, or remove these references and replace them with relevant current references. Any changes to the reference list should be mentioned in the rebuttal letter that accompanies your revised manuscript. If you need to cite a retracted article, indicate the article’s retracted status in the References list and also include a citation and full reference for the retraction notice.

- **Author Response**:
- Thank you for your comment. We have gone through our reference list to ensure everything is correct. No articles we cited have been retracted.

Additional Editor Comments (if provided):

This study highlights a significant public health issue by identifying hazardous lead levels in traditional eyeliners used by refugee and immigrant communities. The research underscores the need for stronger regulations and community education to reduce exposure and protect vulnerable populations, thereby helping to decrease health disparities.

Reviewers:

Reviewer 1:

Minor suggestions for improvement:
If only other reviewers requested too, some method details in the supplementary materials could be moved to the main text.

- **Author Response**:
- Thank you for your comment. The other reviewer requested more information on the reason we had multiple analytical methods for measuring lead in eyeliners. We added this information on line 205. In addition, we have added more information on sample preparation that took place before the eyeliners were sent to the laboratories for testing. This information has been added to lines 180 and 194.

Although it is not final form, in the reviewer copy of the manuscript, the figure 1 is in low resolution. Figure 1 could benefit from higher resolution images.

- **Author Response**:
- Thank you for your comment. We have edited the photo so that it is 300 dpi, is 2250 pixels x 1590 pixels, and is in .tif format. This should improve the resolution.

Reviewer #2: The manuscript is well-written, interesting to read, used rigorous statistical methods for data analysis etc. While the presence of lead in cosmetic products, especially eyelines has been discussed in the literature for some time, the comprehensive approach, using both qualitative and quantitate methods makes this research a valuable contribution to the body of research.

Some minor comments:

Line 36 - "aged" instead of "ages"

- **Author Response**:
- We fixed this, thank you for your keen eye.

Line 174 - "eyeliners that were suspected to contain lead" - please provide details on why they were suspected, on what basis?

- **Author Response**:
- Thank you for your comment. Eyeliners labeled “kajal”, “kohl”, and “surma” were suspected to contain lead based on prior data gathered during in-home lead investigations and community product lead testing events. These events consistently identified traditional eyeliners with high lead concentrations that were labeled as “kajal”, “kohl”, and “surma”.
- This clarification has been added to the text at line 178 (2.3: Acquisition of eyeliner samples).

Section 2.5 Sample analysis - The authors have used 3 different types of chemical analysis of the samples - ICP MS, ICP OES and GF AAS. What was the rationale for using 3 different types? Especially that the methods have various RLs and it ended up being a limitation for the research.

- **Author Response**:
- Thank you for your comment. The employment of different methods arose from the use of pre-existing data collected by Public Health - Seattle and King County (PHSKC) during in-home lead investigations and community product lead testing events. However, once our study commenced, all eyeliner samples were analyzed exclusively using GFAA because NVL Laboratories, Inc. (Seattle, WA), the laboratory doing most of the eyeliner analysis, was able to achieve the lowest reporting limits (RL) with GFAA. In contrast, the eyeliners tested before our study were analyzed using different methods as different PHSKC programs submitted samples for testing without a standardized analytical protocol in place.
- This clarification has been added into the text at line 205 (2.5: Sample analysis). We also discuss the differences in more detail in the paragraph beginning at line 433 (4.5: Strengths and limitations of the study).

Line 214 - here and in several other places the authors speak about the RLs that the lead concentrations in samples were checked against. They provide different RLs in Table 8 and report the percentage of samples
Thank you.

- **Author Response**:
- Thank you for your comment, it highlights the confusion of including “Reporting limits” and “Regulatory limits” in the manuscript, with the same acronyms. We changed “Regulatory limits’ for “Safety guidelines” in Table 8 and throughout the manuscript to prevent this confusion. We have also added notes in Table 3 and Table 4 to clarify the differences in reporting limits (RL) are due to the different analytical methods.
